# Detection of *Felis catus* Gammaherpesvirus 1 in Cats, but not Dogs, in Türkiye: Prevalence and Risk Factors

**DOI:** 10.1002/vms3.71075

**Published:** 2026-07-06

**Authors:** Murat Şevik

**Affiliations:** ^1^ Department of Virology, Veterinary Faculty Necmettin Erbakan University, Ereğli Konya Türkiye

**Keywords:** cat, dog, *Felis catus* gammaherpesvirus 1, genetic characterisation, risk factors

## Abstract

**Background:**

The first gammaherpesvirus to be identified in cats, *Felis catus* gammaherpesvirus 1 (FcaGHV1), has been identified in different countries worldwide. However, there are limited studies on FcaGHV1 infection in Türkiye.

**Objectives:**

The current study was carried out to determine prevalence of FcaGHV1 in Turkish domestic cats and dogs and to investigate potential risk factors that are associated with the detection of FcaGHV1.

**Methods:**

Whole blood samples were collected from cats (*n* = 54) and dogs (*n* = 44). The glycoprotein B (gB) gene‐based TaqMan real‐time PCR assay was utilised to determine the presence of FcaGHV1‐specific DNA. Furthermore, the gB gene region of samples that were found positive in the TaqMan real‐time PCR assay was amplified through conventional PCR.

**Results:**

FcaGHV1‐specific DNA was detected in 4 (7.4%, 95% confidence interval 0.4–14.4) of the 54 feline blood samples by TaqMan real‐time PCR. However, FcaGHV1‐specific DNA was not detected in the EDTA‐blood samples from dogs. The field isolates in this study and isolates from domestic cats in Austria, Japan, Panama, Singapore, Switzerland, and the USA were clustered together by the phylogenetic analysis based on gB gene sequences. Analysis of risk factors showed that there was no significant association between the investigated variables (age, sex, breed, health status, neutering status and outdoor access) and FcaGHV1 detection.

**Conclusions:**

According to the current study, FcaGHV1 circulates in domestic cats in Türkiye. Furthermore, the results of this study suggest that virus transmission does not occur between cat and dog species. It is necessary to conduct more research to determine the prevalence of FcaGHV1 in Türkiye and to confirm the risk factors associated with FcaGHV1 detection.

## Graphical abstract

The current study was carried out to determine prevalence of *Felis catus* gammaherpesvirus 1 (FcaGHV1) in Turkish domestic cats and dogs and to investigate potential risk factors that are associated with the detection of FcaGHV1. FcaGHV1‐specific DNA was detected in 4 (7.4%, 95% confidence interval 0.4–14.4) of the 54 feline blood samples by TaqMan real‐time PCR. However, FcaGHV1‐specific DNA was not detected in the EDTA‐blood samples (*n* = 44) from dogs. Furthermore, analysis of risk factors showed that there was no significant association between the investigated variables (age, sex, breed, health status, neutering status and outdoor access) and FcaGHV1 detection.

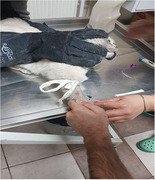

## 1 Introduction

For a long time, it was thought that domestic cats were the natural hosts of a single herpesvirus, felid alphaherpesvirus 1, which causes respiratory and ocular diseases (Gould 2011). In 2014, a new feline herpes virus was identified in the United States, which shares genetic similarities with gammaherpesviruses. It was named *Felis catus* gammaherpesvirus 1 (FcaGHV1) (currently named Percavirus felidgamma1) (Troyer et al. 2014). Studies conducted in various countries have indicated that FcaGHV1 is common in domestic cats worldwide. The detection rate of FcaGHV1 in cats across Europe, Australia, Japan, Brazil, North America, and Singapore has been reported as ranging from 1% to 23.6% (Beatty et al. 2014; Troyer et al. 2014; Ertl et al. 2015; McLuckie et al. 2016; Tateno et al. 2017; Kurissio et al. 2018; Caringella et al. 2019; Novacco et al. 2019).

FcaGHV1 infection has been linked to health status, sex, age, and coinfections with other microorganisms. Infected cats are more prevalent in adult, male, and intact cats (Beatty et al. 2014; Tateno et al. 2017; Caringella et al. 2019). Furthermore, an association between FcaGHV1 infection and retroviruses, including feline leukaemia virus and feline immunodeficiency virus, has been reported (Tateno et al. 2017; Novacco et al. 2019). Immunocompromised animals tend to develop lymphoproliferative disorders more frequently when they come into contact with gammaherpesviruses (Fujiwara and Nakamura 2020). Although it is associated with reduced survival in cats with high‐grade lymphoma, the pathobiological significance of FcaGHV1 in cats remains unclear (McLuckie et al. 2018).

Gammaherpesviruses (GHVs) are generally host‐specific. However, in situations such as immunosuppression or infection of a non‐adapted host, GHVs can cause disease symptoms in other species than their natural hosts (Kruger et al. 2000; Chiou et al. 2005). For example, cattle are considered the natural host of gammaherpesvirus bovine herpesvirus‐4 (BHV‐4), but BHV‐4 has also been detected lions and cats (Egelhof et al. 1991; Kruger et al. 2000). An in vitro study has demonstrated that BHV‐4 can replicate in human cells, and cytopathic effects and intranuclear inclusion bodies can be observed in these cells (Egyed 1998). Furthermore, the Epstein‐Barr virus, a gammaherpesvirus that infects humans worldwide, has been detected in canine peripheral blood mononuclear cells (Chiou et al. 2005). It has been reported that FcaGHV1 infection is transmitted horizontally (Novacco et al. 2019), and FcaGHV1 can infect feline species other than its natural host. Makundi et al. (2018) reported that FcaGHV1 was transmitted between free‐roaming domestic cats and Tsushima leopard cats due to close interactions. Furthermore, Troyer et al. (2014) discovered that Lynx rufus GHV 1, another gammaherpesvirus, was present in both bobcats and pumas, and they suggested that cross‐species transmission is possible between bobcats and pumas. Based on the findings of Troyer et al. (2014) and Makundi et al. (2018), we hypothesised that the FcaGHV1 virus could be transmitted between cats and dogs due to close interactions. To test this hypothesis, the presence of FcaGHV1 in dogs was investigated in this study.

Furthermore, there is not enough information about the disease's status in Türkiye. According to a literature review, there is only one study in Türkiye so far on FcaGHV1 in cats (Koç and Akkartal 2021), and FcaGHV1 detection and potential risk factors have not been investigated in previous studies in Türkiye. For the first time, potential risk factors that are associated with the detection of FcaGHV1 were investigated in this study in domestic cats in Türkiye.

## 2 Materials and Methods

### 2.1 Sample Collection

This cross‐sectional study was conducted between 2020 and 2021. It was determined that 44 animals should be sampled with a 95% confidence level, an acceptable error rate of 10%, and an expected prevalence of 13.02% (Makundi et al. 2018). The determined number was rounded up to 54 to increase the accuracy of the estimated prevalence among cats. Whole blood samples (EDTA‐treated) were collected from cats (*n* = 54) and dogs (*n* = 44) during clinical examinations in veterinary clinics in Istanbul Province in Türkiye (Table 1). The sampled animals were classified as either healthy or sick based on their medical history and physical examination. Animals that were systemically healthy were included in the healthy animal classification, while those that were systemically unwell were included in the sick animal classification. For each animal, data regarding sex, age (adult, age >3 years; young, age ≤3 years), breed (pure or mixed), health status, neutering status and outdoor access were collected.

**TABLE 1:** Univariable logistic regression analysis for risk factors associated with FcaGHV1 detection.

Variables	Categories	Prevalence of FcaGHV1	OR	(95% CI)	*p* value
Age (years)	3 <	2/11 (18.2%)	4.56	0.56–36.78	0.18
	≤ 3	2/43 (4.7%)			
Sex	Male	2/26 (7.7%)	1.08	0.14–8.31	1.00
	Female	2/28 (7.1%)			
Breed	Pure	1/12 (8.3%)	1.18	0.11–12.52	1.00
	Mixed	3/42 (7.1%)			
Healthy status	Healthy	2/8 (25%)	7.33	0.86–62.18	0.10
	Sick	2/46 (4.3%)			
Neutering status	Yes	1/8 (12.5%)	2.05	0.19–22.57	0.48
	No	3/46 (6.5%)			
Outdoor access	Yes	1/10 (10%)	1.52	0.14–16.33	0.57
	No	3/44 (6.8%)			

Abbreviations: CI, confidence interval; OR, odds ratio.

### 2.2 Total Nucleic Acid Extraction

According to reports, the buffy coat of blood contains high concentrations of nucleic acids specific to herpesviruses (Marenzoni et al. 2018). Therefore, EDTA‐treated whole blood samples were centrifuged at 4°C at 2000 × *g* for 10 min to obtain the buffy coat. A commercial kit (High Pure Viral Nucleic Acid Kit, Roche, Germany) was used to extract total nucleic acids from buffy coat samples. After the extraction process, the nucleic acid samples were stored at –80°C until the analysis. To check for cross‐contamination during extraction, a negative control consisting of phosphate‐buffered saline was used for extraction at a rate of 1 in every 10 samples.

### 2.3 FcaGHV1 Detection by TaqMan Real‐Time PCR

The glycoprotein B (gB) gene‐based TaqMan real‐time PCR assay was utilised to determine the presence of FcaGHV1‐specific DNA. The primers and probe described by Troyer et al. (2014) were used to detect the gB gene of FcaGHV1. The TaqMan real‐time PCR reaction mix was prepared using a commercially available kit (Taq DNA Polymerase, Thermo Fisher Scientific, USA) in a final volume of a 25µl mix, which contained 5µl DNA, 0.2µM probe, and 0.4 µM of each primer. The LightCycler 2.0 instrument (Roche, Germany) was utilised to perform the TaqMan real‐time PCR assay. Amplification conditions were as follows: 95°C for 3 min followed by 45 cycles at 95°C for 5 s and 60°C for 30 s. Samples were considered positive if the cycles’ threshold was ≤ 35 (Hill et al. 2024), and nuclease‐free water was used to serve as a negative control in the TaqMan real‐time PCR assay.

### 2.4 gB Gene‐Based Conventional PCR and Sequencing

The gB gene region of positive samples from the TaqMan real‐time PCR assay was amplified by using conventional PCR with primer pairs described by Beatty et al. (2014). The PCR reaction mix was prepared using a commercially available kit (Taq DNA Polymerase, Thermo Fisher Scientific, USA) in a final volume of a 50µl mix, which contained 5µl DNA and 0.4µM of each primer. Amplification conditions were as follows: 94°C for 2 min, followed by 35 cycles at 94°C for 30 s, 57°C for 30 s and 72°C for 30 s, followed by an extension step at 72°C for 5 min. A 1.5% agarose gel that was stained with a commercial gel (GelRed, Biotium Inc., USA) was used to analyse the PCR products. Amplicons (360 bp) were purified from the gel using a commercially available kit (QIAquick Gel Extraction Kit, Qiagen, Germany). Purified DNA samples were sequenced bidirectionally by the Kurul Laboratory (Konya, Türkiye).

Sequences of the gB gene were analysed using BioEdit software (version 7.0.5.3) (Hall 1999). The gB gene consensus sequences were compared for similarity with those in GenBank. Maximum likelihood phylogenetic analysis was conducted using the Kimura 2‐parameter substitution model applied in MEGA11 software with additional sequences retrieved from GenBank. The confidence interval was calculated using bootstrapping with 1000 replicates (Tamura et al. 2021). The gB sequences from this study were deposited in GenBank under accession numbers MW798984, MW798985, PZ033187, and PZ033188.

### 2.5 Statistical Analyses

The statistical analysis was done using SPSS software (version 22, Chicago, USA). The apparent prevalence of FcaGHV1 and its confidence intervals were determined using the EpiInfo 7.2 software. The relationship between FcaGHV1 positivity and potential risk factors was assessed using data collected. First, to confirm the association of independent variables and FcaGHV1 status, a univariable logistic regression analysis was used. The independent variables that had *p* values ≤ 0.2 in the univariable logistic regression analysis were included in the multivariable logistic regression analysis. The final model with the smallest Akaike information criterion was chosen using a manual backward selection method. The Cramer's V and Phi tests were used to check the collinearity of independent variables. In the final model, significant risk factors for FcaGHV1 infection were identified by variables with *p* ≤ 0.05.

## 3 Results

### 3.1 Prevalence of FcaGHV1 and Risk Factors Associated With FcaGHV1 Detection

FcaGHV1‐specific DNA was detected in 4 (7.4%, 95% confidence interval 0.4–14.4) of the 54 feline blood samples. However, FcaGHV1‐specific DNA was not detected in the EDTA‐blood samples (*n* = 44) from dogs.

Cats were categorised into two groups depending on their age: young cats, age ≤3 years old (*n* = 43/54), and adult cats, age >3 years old (*n* = 11/54). The reason cats were categorised into two groups depending on their age (3 years) is that cats older than 3 years are reported to be more susceptible to being positive for FcaGHV1 (Novacco et al. 2019). The age range of the FcaGHV1‐infected cats in this study was between 8 months and 5.5 years.

Results of univariable logistic regression analysis revealed that age and health status (*p* values ≤ 0.2) were significantly associated with FcaGHV1 positivity. The odds of adult cats being FcaGHV1‐positive were 4.56 times higher than the odds for young cats (*p*  =  0.18). Furthermore, the odds of a FcaGHV1 positivity was 7.33 times higher in healthy cats than in sick cats (*p*  =  0.10) (Table 1). Analysis of significant variables with *p* ≤ 0.2, in the univariable logistic regression analysis, was carried out using multivariable logistic regression analysis. There was no significant interaction between the variables, and no confounder was identified. Furthermore, there was no significant association found between age, health status and FcaGHV1 detection in the final model (Table 2).

**TABLE 2:** Multivariable logistic regression results on potential risk factors for FcaGHV1 infection.

Variables	Categories	*B* ^a^	SE^b^	OR (95% CI)	*p* value
Age (years)	≤ 3^c^				
	3 <	1.51	1.06	4.55 (0.56–36.77)	0.15
Healthy status	Sick^c^				
	Healthy	1.99	1.09	7.33 (0.86–62.17)	0.68

^a^ Logistic regression coefficient.

^b^ Standard error.

^c^ Reference category.

### 3.2 Sequence and Phylogenetic Analyses of FcaGHV1 Field Isolates

**FIGURE 1:**
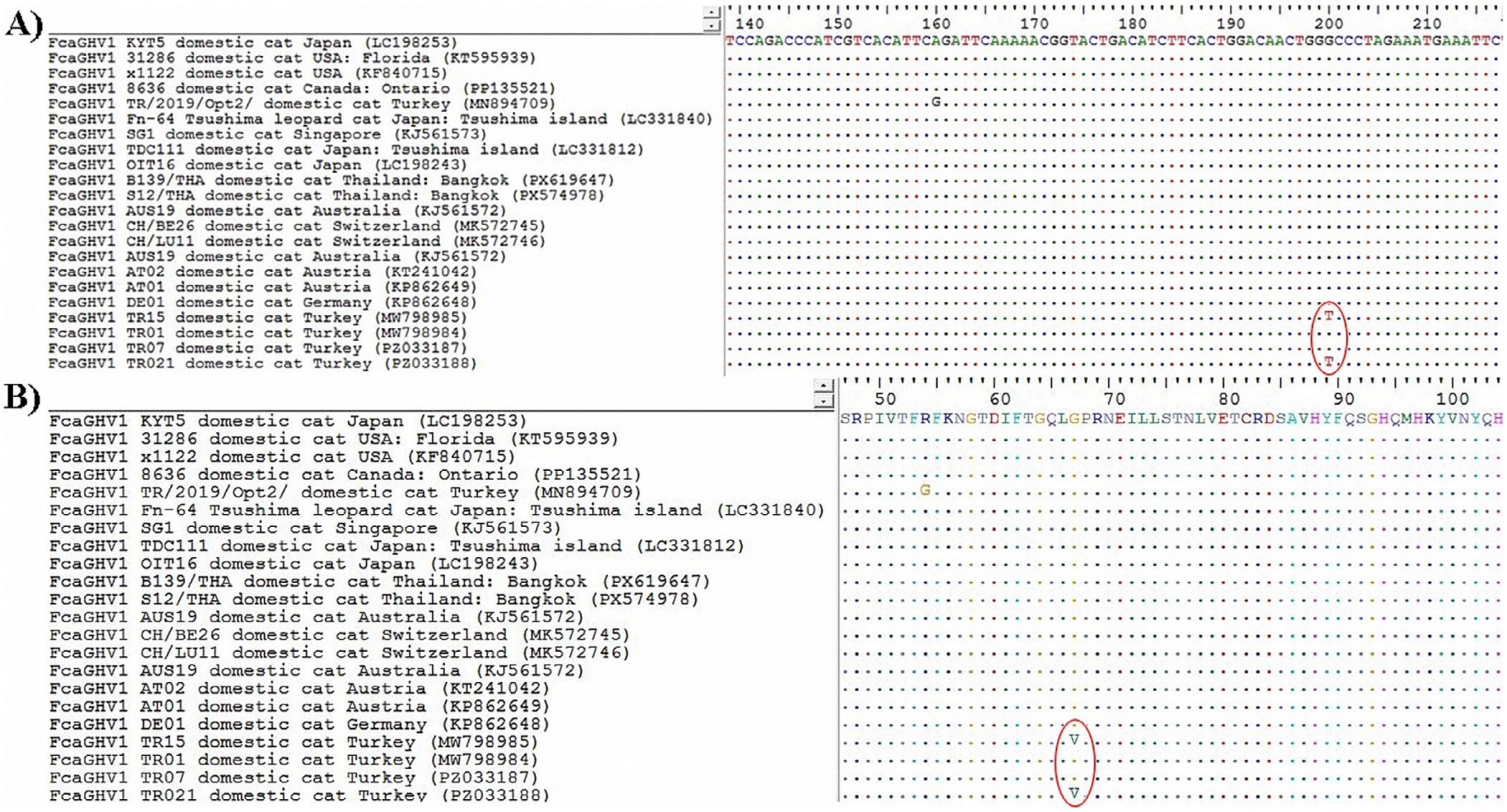
The alignment of FcaGHV1 gB nucleotides (A) and amino acids (B). Alignments were carried out using the ClustalW algorithm that is incorporated in BioEdit software version 7.0.5.3.

Based on the gB gene sequence analysis, the nucleotide homology between the four isolates in this study was between 99.6% and 100%, while the similarity to previously characterised FcaGHV1 isolates was between 98.9% and 100%. Furthermore, four FcaGHV1 isolates from this study had high nucleotide homology (98.9% to 99.3%) with previously characterised Turkish isolates (GenBank MN894708 and MN894709). Two isolates (TR01 and TR07) had identical sequences, but the TR15 and TR021 isolates were different from the others due to a nucleotide mutation (non‐synonymous substitution) at nucleotide 200 of the published complete FcaGHV1 gB sequence (GenBank LC198253) (Figure 1A); glycine is replaced with valine (Figure 1B).

The amino acid identity of the four isolates in this study was between 98.9% and 100%, while the amino acid identity of previously characterised FcaGHV1 isolates was between 97.9% and 100%. Additionally, the four isolates from this study had a 97.9% to 98.9% level of identity when deduced amino acid sequences were compared to previously identified Turkish isolates.

**FIGURE 2:**
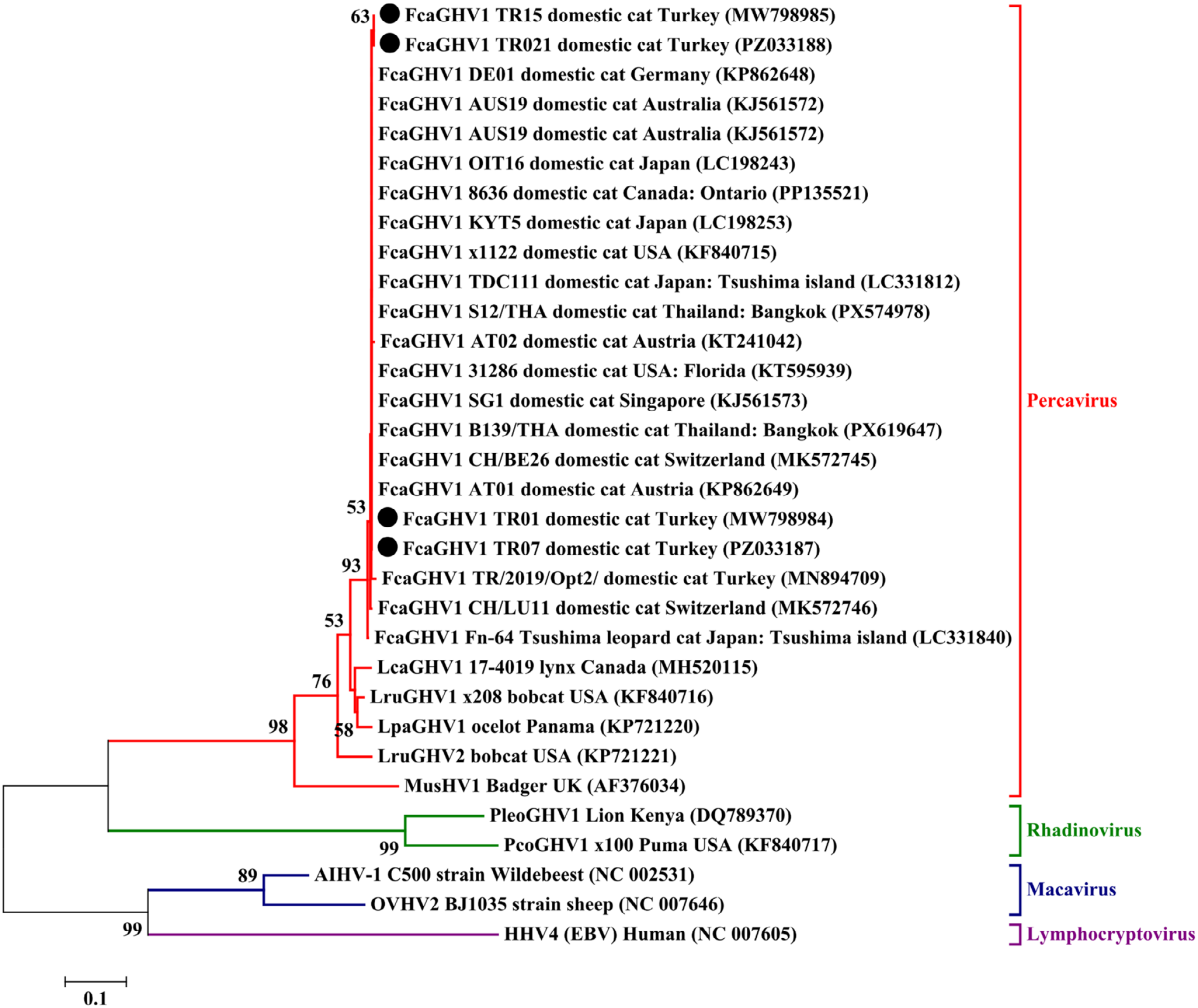
Maximum likelihood phylogenetic tree based on partial gB gene sequences of gammaherpesviruses. A round black spot (●) is used to mark the sample sequences in this study, and bootstrap values higher than 50% were reported. The names of viruses are as follows: LruGHV1: *Lynx rufus* gammaherpesvirus 1, LpaGHV1: *Leopardus pardalis* gammaherpesvirus 1, LcaGHV1: *Lynx canadensis* gammaherpesvirus 1, MusHV1: Mustelid gammaherpesvirus 1, AlHV1: Alcelaphine gammaherpesvirus 1, OvHV2: Ovine gammaherpesvirus 2, PleoGHV1: *Panthera leo* gammaherpesvirus 1, PcoGHV1: *Puma concolor* gammaherpesvirus 1, HHV4: Human gammaherpesvirus 4.

The field isolates in this study and isolates from domestic cats, ocelots, and bobcats in Australia, Austria, Germany, Japan, Panama, Singapore, Switzerland, the USA, and the UK were clustered together in the *Percavirus* genus by the phylogenetic analysis based on gB gene sequences. Furthermore, the FcaGHV1 sequences were grouped in a separate cluster from the other felid GHVs of bobcat, lynx, and ocelot (Figure 2).

## 4 Discussion

The relationship between FcaGHV1 DNAemia and potential risk factors in domestic cats in Türkiye has been investigated by this study for the first time. This study shows that the prevalence of FcaGHV1 was 7.4% (95% CI = 0.4–14.4) in domestic cats in Istanbul Province in Türkiye, which is higher than observed in Balıkesir and Aydın Provinces in Türkiye, where a prevalence of 4.4% was reported (Koç and Akkartal 2021). The difference between the FcaGHV1 rate (7.4%) in this study and the FcaGHV1 rate (4.4%) previously detected in Türkiye can be explained by the sampling strategy (unlike the study by Koç and Akkartal (2021), which only examined cats with ocular disorders, this study examined both healthy and sick cats), the geographic location where the study was conducted and the differences in the cat population in the studied regions (samples in this study were collected from İstanbul Province with a higher cat density than the study of Koç and Akkartal (2021), which was conducted in Balıkesir and Aydın Provinces), and the higher sensitivity of the TaqMan real‐time PCR assay used in this study compared to the conventional PCR method used by Koç and Akkartal (2021).

FcaGHV1 prevalence in domestic cats (7.4%) in this study is less than that reported in other countries. Following its initial detection in 2014, FcaGHV1 has been detected in cats in epidemiological studies conducted in Brazil, North America, Australia, Singapore, and Europe, with infection rates ranging from 9.6% to 23.6% (Beatty et al. 2014; Troyer et al. 2014; Ertl et al. 2015; McLuckie et al. 2016; Kurissio et al. 2018; Makundi et al. 2018). The reason for the lower prevalence of FcaGHV1 among domestic cats in this study compared to other domestic cats in other countries is unclear. This study used the real‐time PCR protocol that has been used in studies with higher viral prevalence, which reduced concerns about the test's sensitivity (Troyer et al. 2014; McLuckie et al. 2016). The success of detecting the virus in low‐titre samples may have been influenced by the storage of tested samples until the analysis. The differences in FcaGHV1 prevalence between countries can be attributed to factors like the geographic differences, the age of the sampled animals, the number of samples examined and the presence of co‐infections. Furthermore, it should be noted that samples used in the current study were obtained from cats during routine clinical examinations at veterinary clinics, not collected randomly from a population of both healthy and infected cats. This could have led to a lower frequency rate, and the results of the current study could not accurately reflect the actual prevalence of cats infected with FcaGHV1 in Türkiye.

The age range of the cats examined in this study ranged from 1 month to 186 months, and the youngest one found to be infected with FcaGHV1 was 8 months old. This result is consistent with a study indicating that cats can become infected at the age of 2 months. Tse et al. (2018) investigated the prevalence of FcaGHV1 infection in cats ranging in age from 2 weeks to 14 years and reported that FcaGHV1 was not detected in cats younger than 2 months.

This study examined the potential risk factors that could be related to FcaGHV1 infection in Turkish domestic cats. Despite the higher proportion of FcaGHV1 infection among males, adults, and purebred cats compared to females, young, and mixed‐breed cats (Table 1), FcaGHV1 detection was not found to have a significant correlation with age, sex, or breed (Table 2). A similar result was obtained in a study conducted by Beatty et al. (2014) on domestic cats in Singapore, and they reported that FcaGHV1 status was not associated with sex or age. Troyer et al. (2014) reported that sex did not pose a risk for gammaherpesvirus infection in wild cats. A study conducted by Caringella et al. (2019) in Italy reported that age was not considered a risk factor for FcaGHV1 infection. Furthermore, sex and breed were not found to be risk factors for infection in a study conducted by Tateno et al. (2017) in Japan. However, different studies have reported a higher prevalence of FcaGHV1 infection in male and older cats compared to younger ones (Troyer et al. 2014; Ertl et al. 2015; Kurissio et al. 2018; Novacco et al. 2019). According to reports, the chance of becoming infected with FcaGHV1 increases with age, and male cats are more likely to get infected due to their more aggressive nature, which increases their risk of exposure to the virus during fighting and also due to the effect of hormonal differences between female and male cats on host immunity (Troyer et al. 2014; Ertl et al. 2015; Beatty et al. 2019; Novacco et al. 2019). Possible explanations for the difference in results between the current study and other studies may be the variations in sample size and the distribution of age and sex among the sampled animals. In this study, the majority of cats tested were young (*n* = 43/54). The outcome may be different if studies involve a larger number of adult cats.

The current study's results are in accordance with those of previous studies (Ertl et al. 2015; Caringella et al. 2019; Novacco et al. 2019) and indicate that neutering status and outdoor access do not pose a risk for FcaGHV1 infection (Table 1).

In this study, FcaGHV1 was detected in both sick (*n* = 2) and healthy (*n* = 2) cats. The clinical signs that were observed in sick cats were fever, loss of appetite, bloody diarrhoea, and vomiting. However, the present did not find any significant correlation between FcaGHV1 detection and the cat's health status (Table 2). Similar results have been obtained in studies conducted in Central Europe and Japan, and it has been reported that FcaGHV1 infection is not the cause of poor health in cats (Ertl et al. 2015; Tateno et al. 2017; Hill et al. 2024). However, studies conducted in Singapore and Australia have reported that sick cats have a higher prevalence of FcaGHV1 infection than healthy cats (Beatty et al. 2014). There is still uncertainty about the pathogenic potential of FcaGHV1 in cats. Because of the absence of information on the immune status of the cats studied, the pathogenicity of FcaGHV1 was not fully evaluated in this study. To understand the role of FcaGHV1 in feline diseases, there is a need for more research.

Troyer et al. (2014) discovered that Lynx rufus GHV 1, another gammaherpesvirus, was present in both bobcats and pumas, and they suggested that cross‐species transmission is possible between bobcats and pumas. Furthermore, Makundi et al. (2018) reported that FcaGHV1 was transmitted between free‐roaming domestic cats and Tsushima leopard cats due to close interactions. Based on these reports, this study investigated the possibility of horizontal FcaGHV1 transmission between cat and dog species. However, FcaGHV1 DNA was not detected in the blood samples of the dogs based on molecular analysis. This result suggests that virus transmission does not occur between cat and dog species. Further comprehensive studies are thought to be beneficial in investigating whether cross‐species transmission occurs.

Sequence comparison of the 289‐bp sequence in the gB gene revealed high nucleotide homology (98.9% to 100%) between the four isolates in the current study and previously characterised FcaGHV1 isolates. Furthermore, four isolates in the current study were found to be clustered in the same cluster with other gammaherpesviruses in the *Percavirus* genus (Figure 2). The findings of this study suggest that the FcaGHV1 isolates from this study are genetically very similar to those found throughout the world. Comparing longer sequences from multiple genes will provide insight into the extent of regional heterogeneity.

**FIGURE 3:**
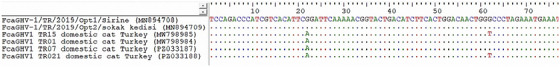
Comparison of partial gB gene nucleotide sequences among four isolates from this study and two previously reported Turkish isolates (TR/2019/Opt1 and TR/2019/Opt2).

Sequence analysis of the gB gene revealed that the isolates of this study differ from the previous Turkish isolates (GenBank MN894708 and MN89479) with two mutations (Figure 3). This result suggests that genomic evolution is occurring slowly despite the FcaGHV1 gB gene being relatively conserved.

The gB gene of FcaGHV1 is responsible for the virus's entry into the cell (Vallbracht et al. 2021). Sequence analysis of FcaGHV1 gB sequences demonstrated that TR15 and TR021 isolates from this study had valine in place of glycine in the gB gene (Figure 1B). Further studies will provide insight into the roles of this mutation in FcaGHV1 pathogenesis.

This study has some limitations. The imbalance between positive and negative cases should be taken into account when interpreting the results of this study. The relatively small number of FcaGHV1‐positive cases limits the statistical power and generalisability of the findings. Additionally, the representativeness of the sample may be limited due to the collection of blood samples from cats and dogs during clinical examinations in veterinary clinics. Also, co‐infections with feline leukaemia virus and feline immunodeficiency virus were not assessed in this study. The understanding of the relationship between these viruses and FcaGHV1 infection could be improved by investigating co‐infection with them.

## 5 Conclusions

According to the current study, FcaGHV1 circulates in domestic cats in Istanbul Province in Türkiye. However, no relationship was found between the investigated variables and FcaGHV1 detection in this study. The present study was limited to one province (Istanbul Province) out of 81 provinces of Türkiye, making it uncertain if the data obtained represents all provinces of Türkiye. Therefore, further studies of FcaGHV1 in Türkiye, including its prevalence and genetic diversity and potential risk factors associated with infection, are required.

## Author Contributions

*Conceptualisation, methodology, analyses, writing – original draft, review and editing*: Murat Şevi.

## Funding

This study has been supported by Hatay Mustafa Kemal University Scientific Research Projects Coordination Unit under project number 20.YL.027.

## Ethics Statement

This study was approved by the Institutional Ethics Committee of the Faculty of Veterinary Medicine, Hatay Mustafa Kemal University (No. 2020/01‐08).

## Conflicts of Interest

The author declares that there is no conflict of interest.

## Data Availability

Upon a justifiable request, the data used in this research can be obtained from the corresponding author.
